# Activation of the NRF2 pathway in *Keap1*-knockdown mice attenuates progression of age-related hearing loss

**DOI:** 10.1038/s41514-020-00053-4

**Published:** 2020-12-14

**Authors:** Tetsuya Oishi, Daisuke Matsumaru, Nao Ota, Hiroshi Kitamura, Tianxiang Zhang, Yohei Honkura, Yukio Katori, Hozumi Motohashi

**Affiliations:** 1 Department of Otolaryngology-Head and Neck Surgery, Tohoku University Graduate School of Medicine, 1-1 Seiryo-machi, Aoba-ku, Sendai, 980-8574 Japan; 2 Department of Gene Expression Regulation, Institute of Development, Aging and Cancer, Tohoku University, 4-1 Seiryo-machi, Aoba-ku, Sendai, 980-8575 Japan

**Keywords:** Transcription, Ageing, Auditory system

## Abstract

Age-related hearing loss (AHL) is a progressive sensorineural hearing loss in elderly people. Although no prevention or treatments have been established for AHL, recent studies have demonstrated that oxidative stress is closely related to pathogenesis of AHL, suggesting that suppression of oxidative stress leads to inhibition of AHL progression. NRF2 is a master transcription factor that regulates various antioxidant proteins and cytoprotection factors. To examine whether NRF2 pathway activation prevents AHL, we used *Keap1*-knockdown (*Keap1*^FA/FA^) mice, in which KEAP1, a negative regulator of NRF2, is decreased, resulting in the elevation of NRF2 activity. We compared 12-month-old *Keap1*^FA/FA^ mice with age-matched wild-type (WT) mice in the same breeding colony. In the *Keap1*^FA/FA^ mice, the expression levels of multiple NRF2 target genes were verified to be significantly higher than the expression levels of these genes in the WT mice. Histological analysis showed that cochlear degeneration at the apical and middle turns was ameliorated in the *Keap1*^FA/FA^ mice. Auditory brainstem response (ABR) thresholds in the *Keap1*^FA/FA^ mice were significantly lower than those in the WT mice, in particular at low–mid frequencies. Immunohistochemical detection of oxidative stress markers suggested that oxidative stress accumulation was attenuated in the *Keap1*^FA/FA^ cochlea. Thus, we concluded that NRF2 pathway activation protects the cochlea from oxidative damage during aging, in particular at the apical and middle turns. KEAP1-inhibiting drugs and phytochemicals are expected to be effective in the prevention of AHL.

## Introduction

Age-related hearing loss (AHL) is the most common sensorineural hearing loss in the elderly and is caused by degenerative and irreversible changes in the inner ear^1–4^. Age-related histological impairment has been reported in hair cells, spiral ganglion neurons (SGNs), the spiral ligament (SL), the stria vascularis (SV), and synaptic connections between hair cells and SGNs^5–9^. Although AHL is thought to be caused by various factors, such as exposure to noise^10,11^, ototoxic chemicals^12^, systemic disorders^13,14^, and genetic predispositions^15–18^, the precise molecular mechanisms of AHL are not well understood. Accordingly, neither effective prevention nor treatments for AHL have been established. As longevity increases worldwide, AHL is becoming an increasingly serious problem that negatively affects the quality of life in the aged population.

Recent studies have suggested that reactive oxygen species (ROS) are closely related to the pathogenesis of AHL^19,20^. ROS are mainly produced by NADPH oxidases and mitochondrial respiration in the cochlea^21,22^. Excessive ROS production or decreased antioxidant capacity induces oxidative damage in the cochlea^23–25^. Although a few antioxidant reagents have been shown to protect the cochlea by decreasing ROS levels^26–29^, no drugs have ever been shown to prevent the progression of AHL.

The transcription factor NRF2 is a master regulator of various detoxifying and antioxidant genes, and is systemically activated in response to oxidative and electrophilic stress^30,31^. Under normal conditions, NRF2 is ubiquitinated by the KEAP1-CUL3 ubiquitin E3 ligase complex in the cytoplasm, resulting in the NRF2 protein degradation through the proteasome. Upon exposure to ROS or electrophiles, which inactivate KEAP1, NRF2 is stabilized and subsequently translocated into the nucleus where it activates many cytoprotective genes, including *NAD(P):quinone oxidoreductase 1* (*Nqo1*), *Thioredoxin reductase 1* (*Txnrd1*), *Glutamate-cysteine ligase, catalytic subunit* (*Gclc*), and *Glutamate-cysteine ligase, modifier subunit* (*Gclm*), by binding to antioxidant response element (GCnnn^G^/_C_TCA^T^/_C_). In addition, recent studies clarified that NRF2 exerts potent anti-inflammatory functions^32,33^. Appropriate activation of NRF2 has been shown beneficial for our health preventing and alleviating various pathological conditions, such as ischemia-reperfusion injury, neurodegenerative diseases, and chronic inflammation^30,31,34^. Regarding the protective role of NRF2 in the cochlea, we previously reported that noise-induced hearing loss (NIHL) was exacerbated in *Nrf2*-knockout mice and prevented by pretreatment of NRF2 inducers^35^. *Gsta4*, one of the typical NRF2 target genes, was shown to play a critical role in the protection from drug ototoxicity in female mice^36^. In addition, AHL progression was reported to be accelerated in *Nrf2*-knockout mice. The numbers of hair cells and spiral ganglion cells were reduced earlier in *Nrf2*-knockout mice than they were in wild-type (WT) mice^37^. Thus, endogenous NRF2 is regarded as necessary for resisting the progression of age-related pathology of the inner ear.

In the current study, we investigated whether NRF2 pathway activation suppresses AHL progression. To this end, we examined *Keap1*-knockdown (*Keap1*^FA/FA^) mice, in which NRF2 pathway is systemically activated due to the decreased expression of *Keap1*^38,39^. We compared congenic *Keap1*^FA/FA^ mice with WT control mice on a C57BL/6 genetic background at the ages of 2, 5, and 12 months. As the C57BL/6 strain exhibits early onset of AHL due to a single-nucleotide polymorphism (SNP) of *Cdh23* gene, we evaluated the mice at 12 months of age, which is generally considered as middle age^40–42^. As expected, the 12-month-old *Keap1*^FA/FA^ mice were well protected from oxidative damage and degenerative alterations of the inner ear, and retained better hearing ability than the WT mice at the same age. These results strongly suggest that chemicals and stimuli that activate NRF2 pathway, or KEAP1 inhibitors, are effective for the prevention of AHL.

## Results

### AHL in the WT C57BL/6 mice

**Fig. 1:**
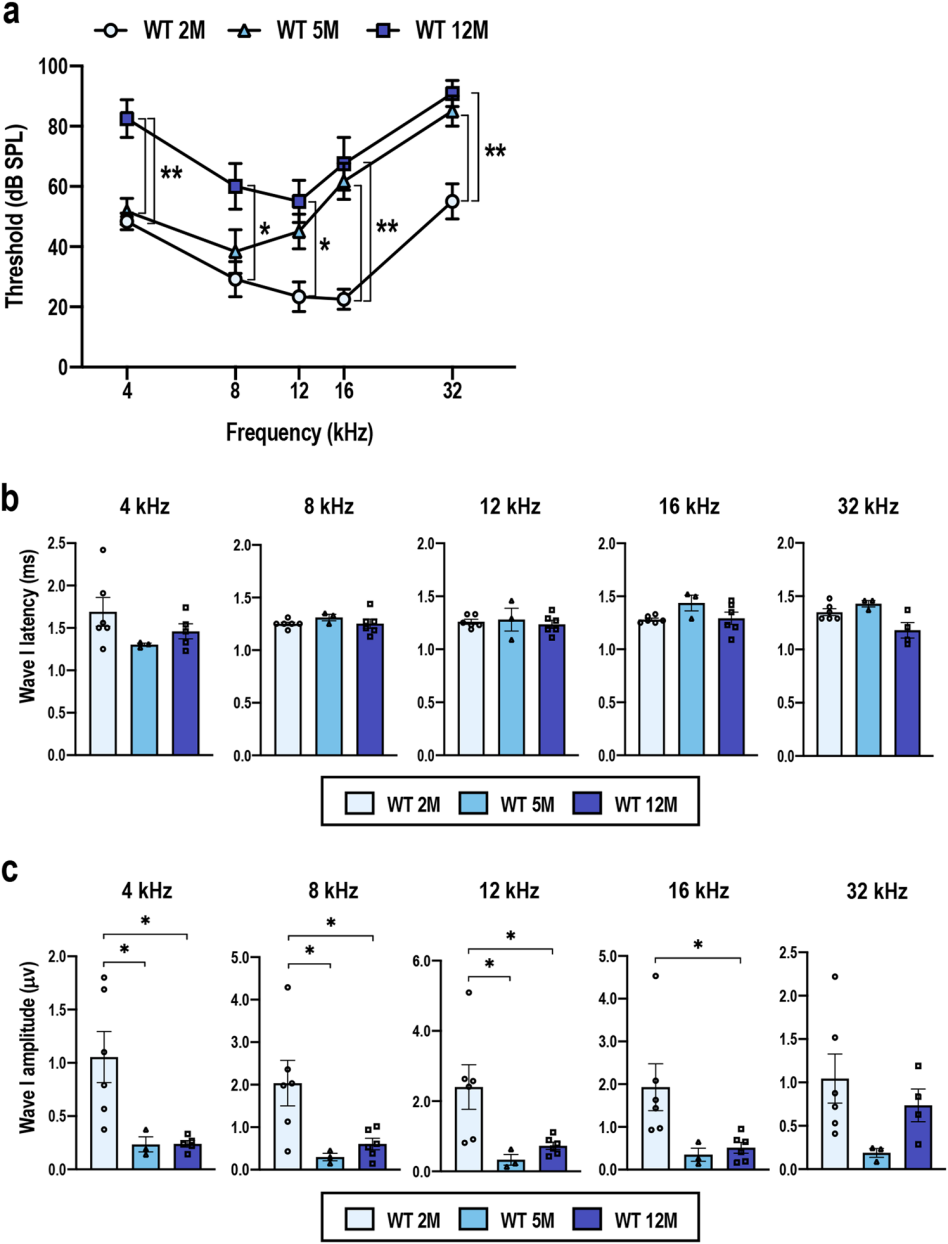
Progression of hearing impairment in the WT C57BL/6 mice during aging. ABR thresholds (**a**), ABR wave I latencies (**b**), and ABR wave I amplitudes (**c**) of the wild-type (WT) C57BL/6 mice at the age of 2, 5, and 12 months (*n* = 6 for 2-month-old mice, *n* = 3 for 5-month-old mice, and *n* = 6 for 12-month-old mice). The data represent the mean ± SEM. *P < 0.05, **P < 0.01. Two-way analysis of variance (ANOVA) followed by Tukey’s multiple comparison test was applied. As the ABR wave I latencies and amplitudes that were induced by a stimulus intensity of 100 dB SPL were measured, one and two 12-month-old mice that exhibited ABR thresholds above 100 dB SPL at 4 and 32 kHz, respectively, were omitted from the waveform analysis shown in **b** and **c**.

The C57BL/6 mouse is a well-known model of early onset of AHL, and functional and structural impairment of cochlea within 1 year have been documented^42–44^. We first examined AHL progression in the WT C57BL/6 mice by comparing those at 2, 5 and 12 months of age in our breeding colony. Auditory brainstem response (ABR) thresholds were significantly elevated in the 5-month-old mice compared with those in the 2-month-old mice at frequencies of 16 and 32 kHz, respectively (Fig. 1a). Significant elevation of the ABR thresholds at all frequencies from 4 to 32 kHz were observed by 12 months of age (Fig. 1a). These results suggest that the hearing ability became gradually impaired, and that AHL was fully apparent by 12 months of age. To assess the SGN function, we examined latency and amplitude of the ABR wave I induced by a stimulus intensity of 100 dB sound pressure level (SPL). The latencies were comparable irrespective of mouse age (Fig. 1b), whereas the amplitudes were mostly reduced in 5- and 12-month-old mice (Fig. 1c). These results imply that functional impairment of the SGN activity was already started by 5 months of age.

**Fig. 2:**
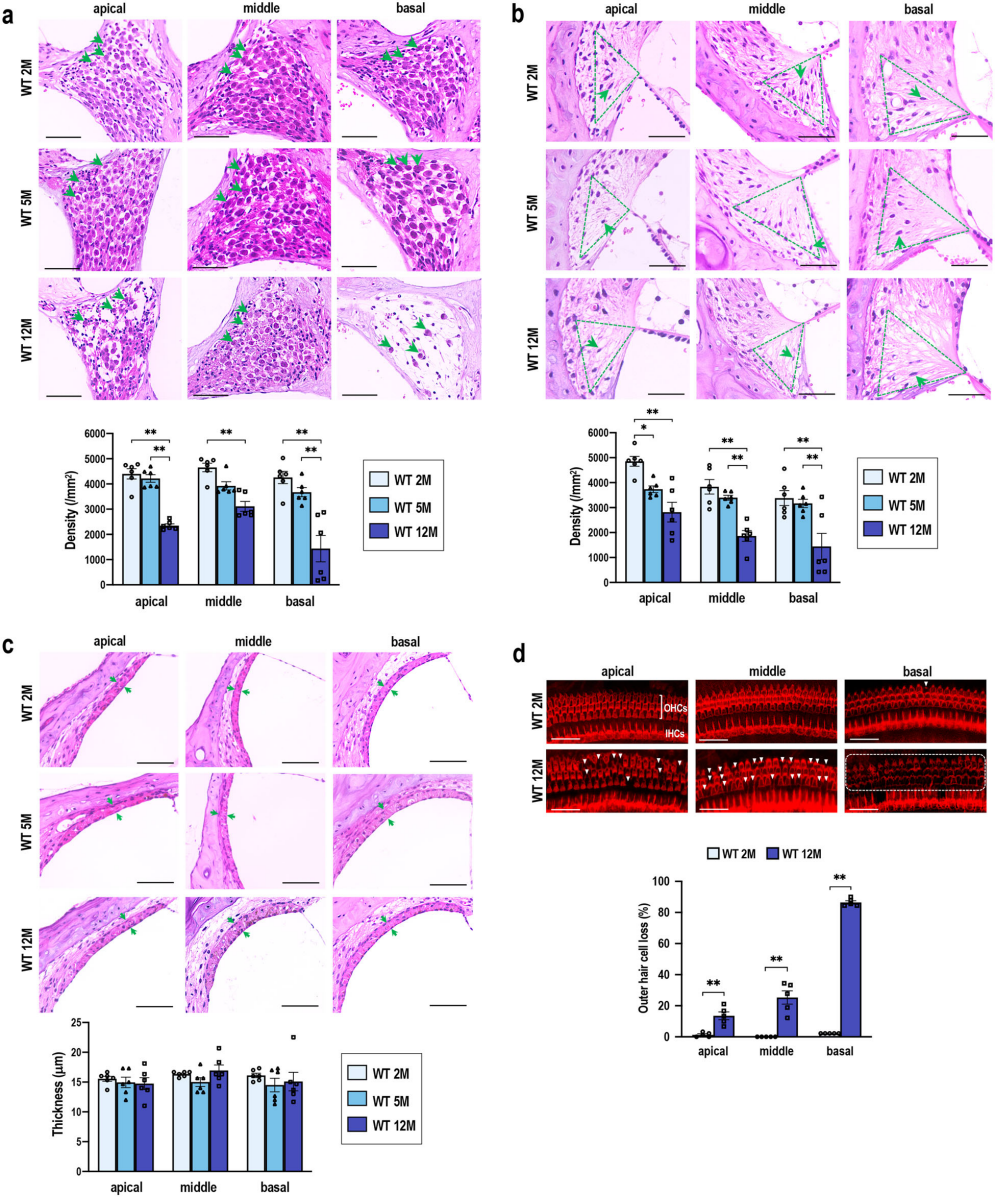
Progression of cochlear degeneration in the WT C57BL/6 mice during aging. **a**–**c** Histological analysis of spiral ganglion neurons (SGNs) (**a**), spiral ligaments (SL) (**b**), and stria vascularis (SV) (**c**) at the apical, middle, and basal turns in the 2-, 5-, and 12-month-old mice (*n* = 6 in each group). SGN density (**a**), type IV fibrocyte density in the SL (**b**), and SV thickness (**c**) were quantified. Green arrows indicate neuronal soma (**a**), fibrocytes (**b**), and SV width (**c**). Areas of type IV fibrocytes are indicated by dashed green triangles. The data represent the mean ± SEM. *P < 0.05, **P < 0.01. Two-way ANOVA followed by Tukey’s multiple comparison test was applied. **d** Images of the surface preparation of the hair cells at 2 and 12 months. Missing outer hair cells (OHCs) are indicated with white arrowheads. Most OHCs in the basal turns at 12 months were lost, as indicated in the area circumscribed by the dashed line. IHCs, inner hair cells. Missing OHCs were quantitatively analyzed by evaluating 90 OHCs at each turn (*n* = 5 in each group). The data represent the mean ± SEM. **P < 0.01. Unpaired two-tailed Student’s *t*-test was applied. Scale bars correspond to 50 μm (**a**–**c**) and 40 μm (**d**).

Histological alterations of the cochleae were apparent in the SGNs and SL at 12 months of age but not in SV (Fig. 2a–c). The SGN density was significantly decreased in the 12-month-old mice compared with that in the 2- and 5-month-old mice at all cochlear turns (Fig. 2a). The density of the SL fibrocytes, in particular those defined as type IV fibrocytes^45^, was lower in the 12-month-old mice than it was in the 2- and 5-month-old mice at all cochlear turns (Fig. 2b). In contrast, the thickness of the SV showed no significant differences among the three groups (Fig. 2c). Surface preparation analysis of the hair cells revealed a dramatic difference between the 2- and 12-month-old mice. Although occasional loss of outer hair cells (OHCs) was observed at only the basal turns in the 2-month-old mice, a high degree of OHC loss was apparent at all the turns in the 12-month-old mice (Fig. 2d). These results indicated that the WT mice had fully developed AHL by 12 months of age, both in functional and structural aspects.

### Cochlear gene expression in WT C57BL/6 mice

**Fig. 3:**
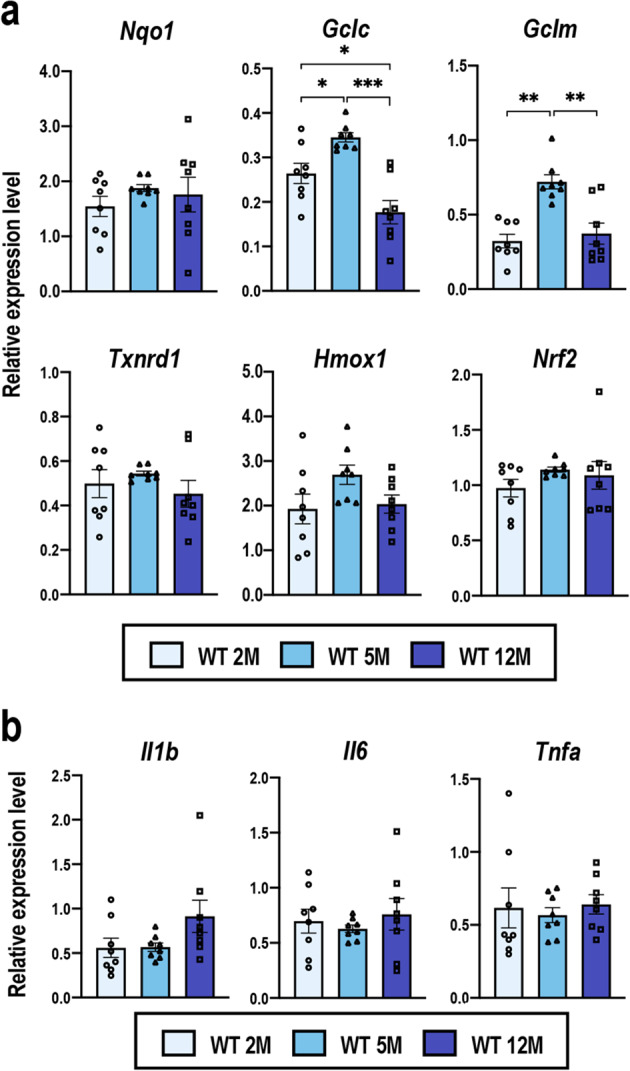
Gene expression in the WT C57BL/6 cochleae during aging. Expression levels of *Nrf2*, NRF2 target genes (**a**), and proinflammatory cytokine genes (**b**) in whole cochleae of the WT C57BL/6 mice at 2, 5, and 12 months of age were examined by quantitative real-time PCR. All the samples were quantified by using the same standard curve and each expression level was normalized to that of the *Hprt* or *Beta-Actin* expression level. Data represent the mean ± SEM (*n* = 8 in each group). *P < 0.05, **P < 0.01, ***P < 0.001. Two-way ANOVA followed by Tukey’s multiple comparison test was applied.

We next compared NRF2 pathway activity in WT mice at 2, 5, and 12 months of age by examining the expression levels of representative NRF2 target genes *Nqo1*, *Gclc*, *Gclm*, *Txnrd1*, and *Hmox1*, as well as *Nrf2*. The expression levels were nearly the same among the three groups and no apparent age-related changes were observed, except for *Gclc* and *Gclm*, which were higher in the 5-month-old cochlea than the rest for unknown reasons (Fig. 3a). As the pathogenesis of NIHL correlates with inflammation^46–48^ and, as previous reports suggested, inflammation also contributes to AHL^49,50^, we additionally investigated the expression of the proinflammatory cytokine genes *Il1b*, *Il6*, and *Tnfa*. However, the expression of these proinflammatory cytokine genes was not significantly different among the three groups (Fig. 3b). These results suggested that either deterioration of NRF2 pathway activity or inner ear inflammation was not directly related to AHL in the WT mice in our experimental setting.

### Comparison of the *Keap1*^FA/FA^ mice and WT mice at 2 months of age

**Fig. 4:**
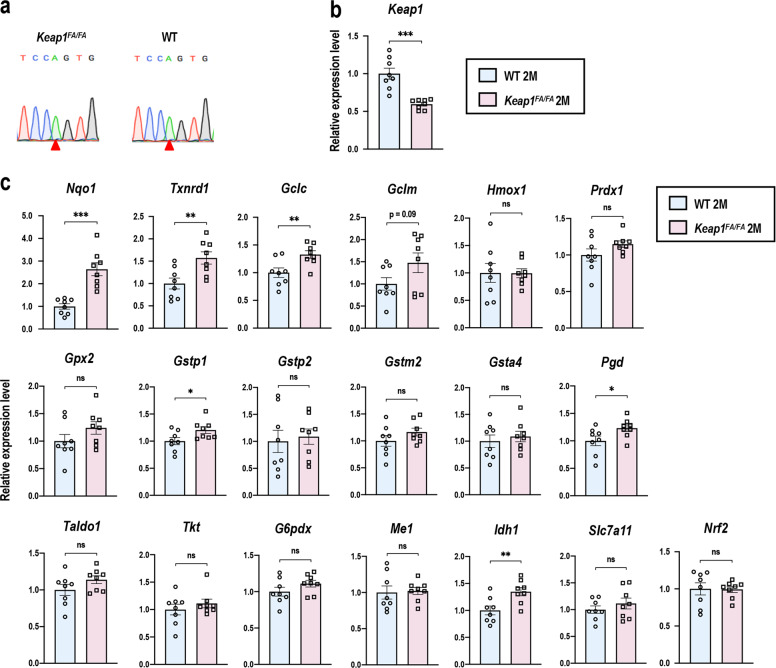
Gene expression in cochleae of the WT and *Keap1*^FA/FA^ mice at 2 months of age. **a** Detection of an SNP in the *Cdh23* gene (*n* = 6 in each group). Red arrowheads indicate the SNP of the *Cdh23*^*753A*^ allele. Expression levels of *Keap1* (**b**) and NRF2 target genes and *Nrf2* (**c**) in the whole cochleae were measured by quantitative real-time PCR (*n* = 8 in each group). All the samples were quantified by using the same standard curve and each expression level was normalized to *Hprt* expression. Average expression levels of WT cochlea are set as 1. Data represent the mean ± SEM. *P < 0.05, **P < 0.01, ***P < 0.001. Unpaired two-tailed Student’s *t*-test was applied. A part of data on the WT mice at 2 months of age are the same as those shown in Fig. 3.

The C57BL/6 strain of mice possesses an SNP in the *Cdh23* gene, *Cdh23*^ahl^ allele, which is thought to cause an early decline in hearing ability in this strain of mice^41,51^. To verify the genotype of *Cdh23* in the *Keap1*^FA/FA^ and WT mice, which had been backcrossed into the C57BL/6 background, we sequenced their tail DNA samples obtained from our breeding colony through random sampling. All the *Keap1*^FA/FA^ and WT mice had the *Cdh23*^ahl^ allele (*Cdh23*^*753A/753A*^) (Fig. 4a).

**Fig. 5:**
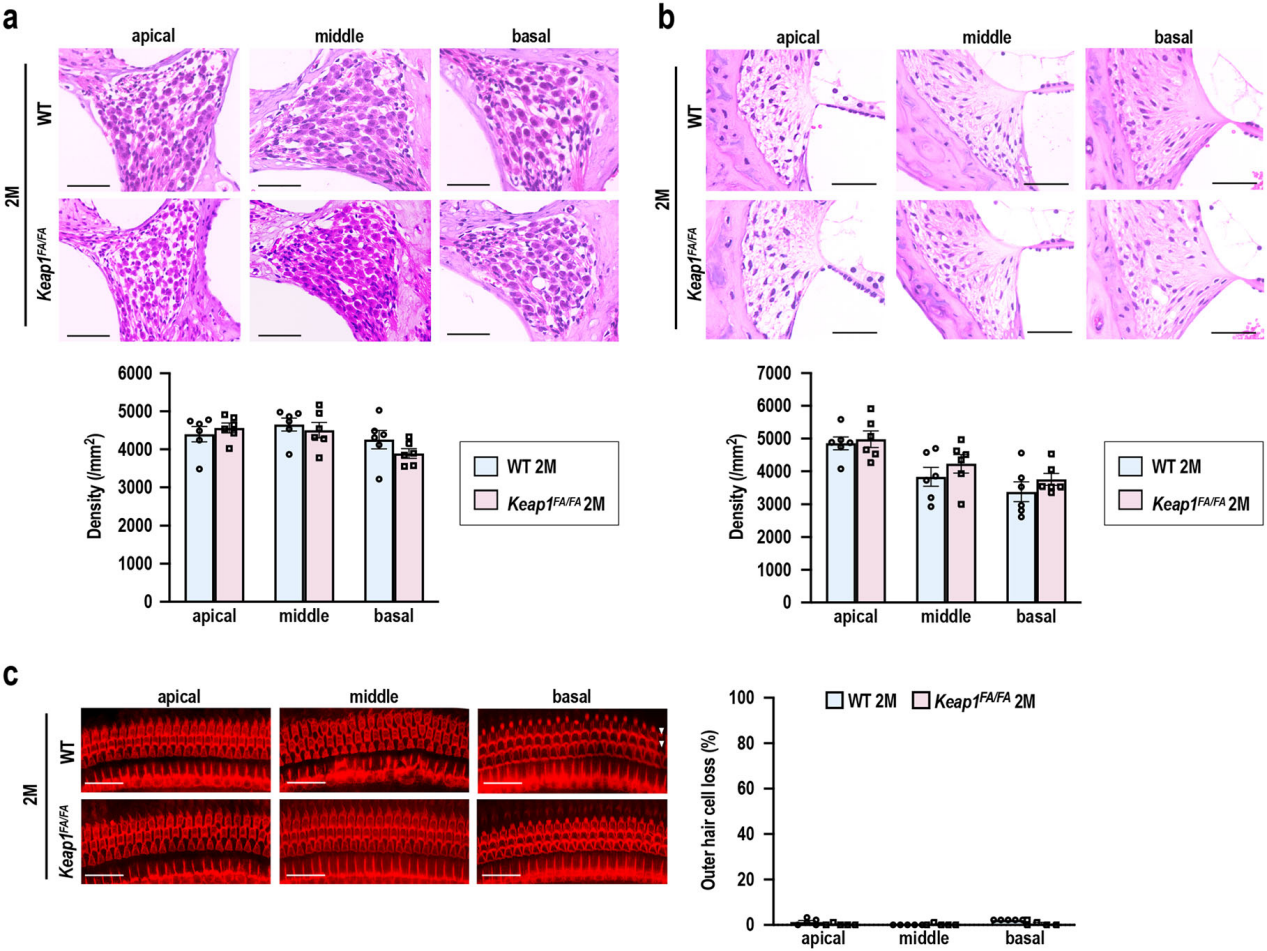
Comparison of cochlear structure in the WT and *Keap1*^FA/FA^ mice at 2 months of age. **a**, **b** Cochlear histology at each turn. The SGN density (**a**) and density of the SL fibrocytes (**b**) are shown (*n* = 6 in each group). **c** Surface preparation images of the hair cells. Missing OHCs are indicated with white arrowheads. Missing OHCs were quantitatively analyzed by evaluating 90 OHCs at each turn (*n* = 5 in each group). Data represent the mean ± SEM. Unpaired two-tailed Student’s *t*-test was applied. Differences between WT and *Keap1*^FA/FA^ mice were not statistically significant. The samples of the WT mice at 2 months of age are the same as those shown in Fig. 2. Scale bars correspond to 50 μm (**a**, **b**) and 40 μm (**c**).

We next characterized the inner ears of the *Keap1*^FA/FA^ mice at 2 months of age. Although the *Keap1*^FA/FA^ mice were shown to exhibit activation of the NRF2 pathway due to reduced expression of the *Keap1* gene in various organs^38,39^, no information was available on the *Keap1* expression status of the cochlea in the *Keap1*^FA/FA^ mice. We found decreased expression of *Keap1* (Fig. 4b) and significantly increased expression of *Nqo1*, *Txnrd1*, *Gclc*, *Gstp1*, *Pgd*, and *Idh1*, 6 out of 18 representative NRF2 target genes (Fig. 4c), in *Keap1*^FA/FA^ cochlea compared with WT cochlea. Expression of *Gclm*, *Prdx1*, and *Gpx2* tended to be increased in *Keap1*^FA/FA^ cochlea, but without reaching statistical significance (Fig. 4c). *Nrf2* mRNA expression were comparable between the two genotypes (Fig. 4c). These results suggested that the NRF2 pathway was activated in the cochlea by decreased expression of *Keap1*, although we could not detect a clear increase in NRF2 protein level in the *Keap1*^FA/FA^ cochlear extracts (data not shown), probably because NRF2 protein accumulation induced by ~50% reduction of *Keap1* mRNA was rather mild. Histological and morphological examination showed that the cochleae were not apparently different between the mice of the two genotypes (Fig. 5a–c).

**Fig. 6:**
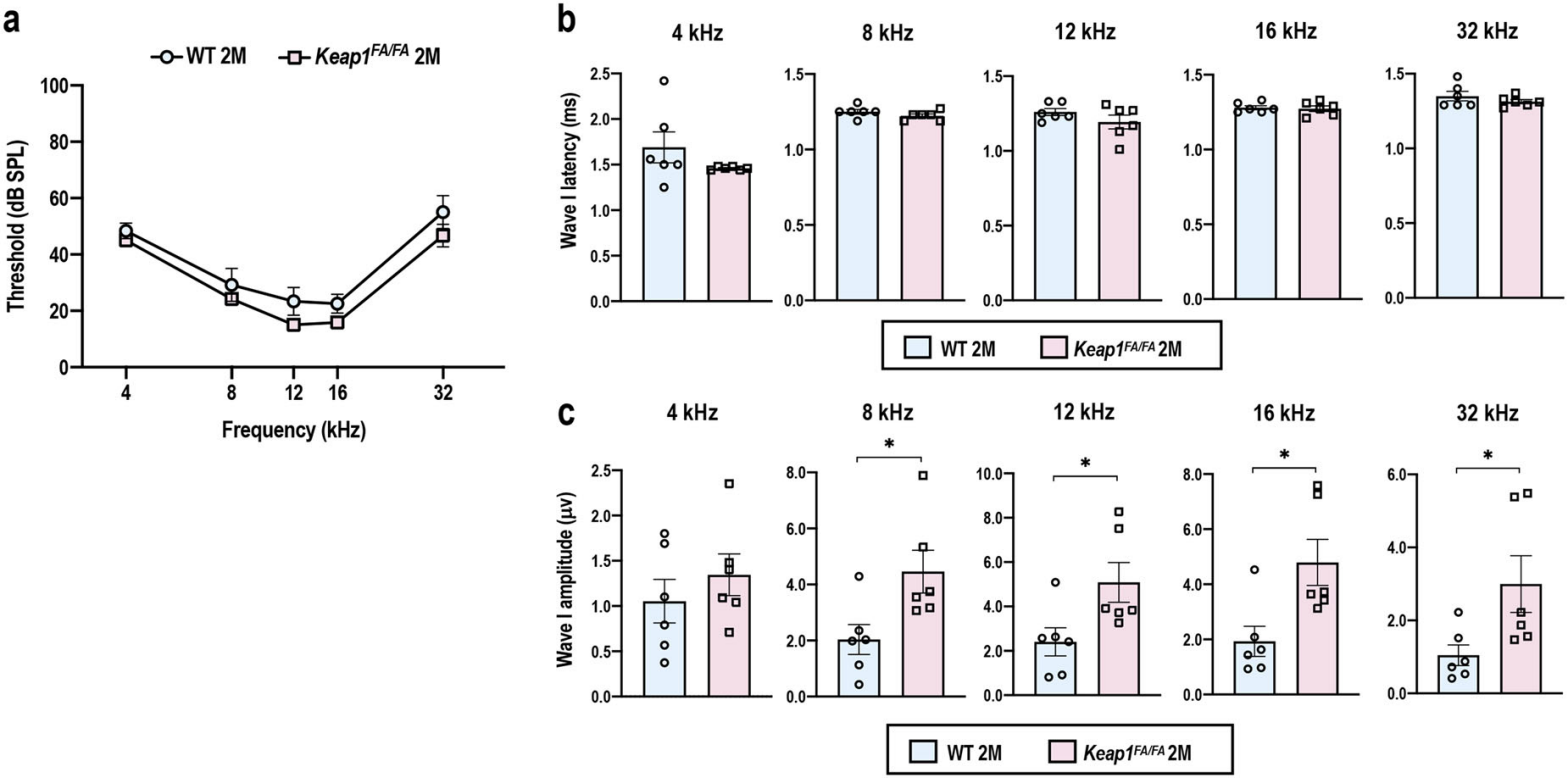
Comparison of ABR of the WT and *Keap1*^FA/FA^ mice at 2 months of age. ABR thresholds (**a**), ABR wave I latencies (**b**), and ABR wave I amplitudes (**c**) of the WT and *Keap1*^FA/FA^ mice at the age of 2 months (*n* = 6). The data represent the mean ± SEM. *P < 0.05. Unpaired two-tailed Student’s *t*-test was applied. The samples of the WT mice at 2 months of age are the same as those shown in Fig. 1.

The ABR thresholds were comparable between *Keap1*^FA/FA^ and WT mice at all frequencies (Fig. 6a). Similarly, the ABR wave I latencies were comparable between the two genotypes (Fig. 6b). Intriguingly, the ABR wave I amplitudes were larger in *Keap1*^FA/FA^ mice than WT mice at 2 months of age (Fig. 6c), implying functional robustness of SGNs in *Keap1*^FA/FA^ cochlea.

### Comparison of the *Keap1*^FA/FA^ mice and WT mice at 5 months of age

**Fig. 7:**
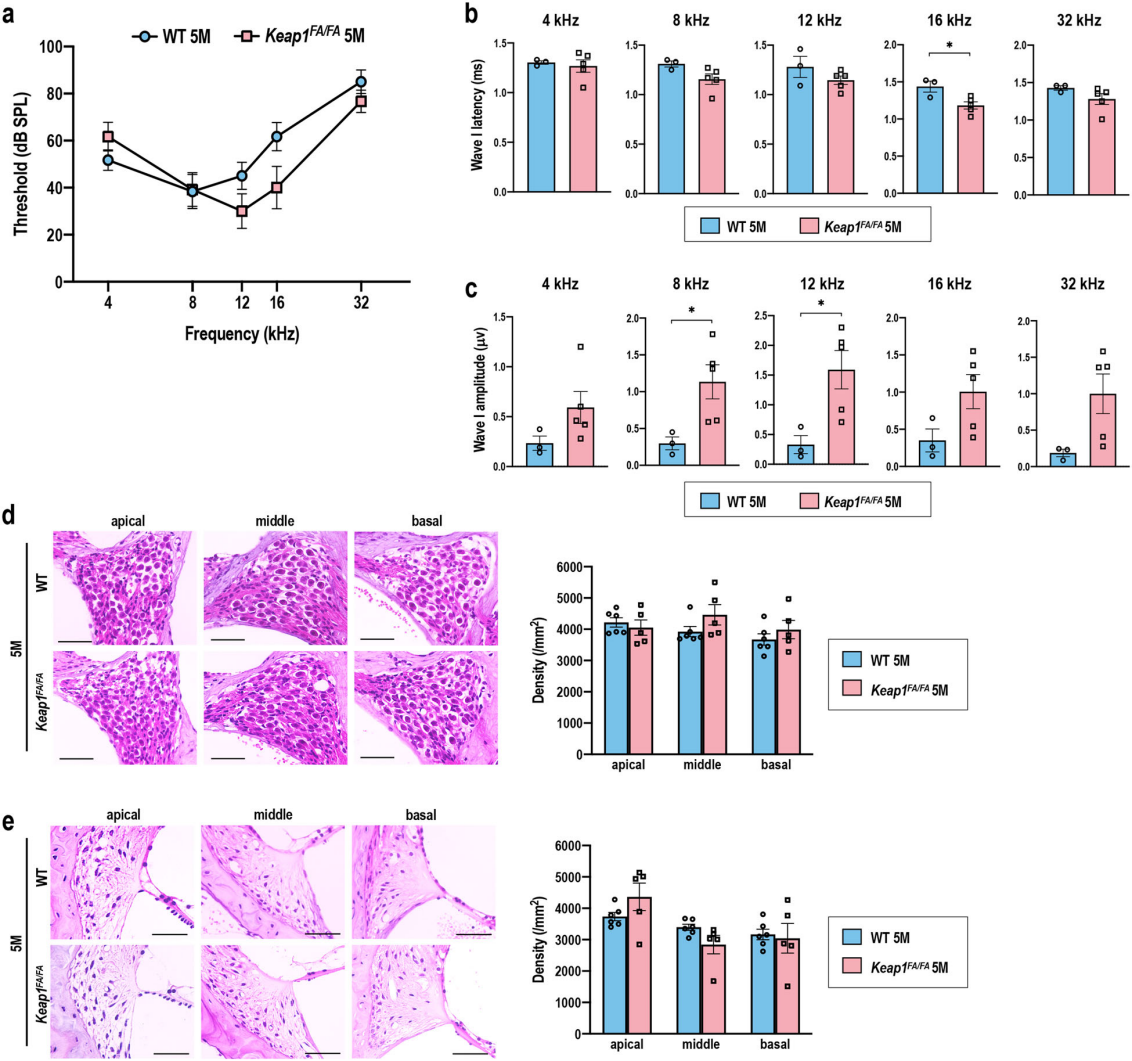
Comparison of ABR and cochleae in the WT and *Keap1*^FA/FA^ mice at 5 months of age. ABR thresholds (**a**), ABR wave I latencies (**b**), and ABR wave I amplitudes (**c**) of the WT and *Keap1*^FA/FA^ mice at the age of 5 months (*n* = 3 for WT mice and *n* = 5 for *Keap1*^FA/FA^ mice). **d**, **e** Cochlear histology at each turn. The SGN density (**d**) and density of the SL fibrocytes (**e**) are shown (*n* = 6 for WT mice and *n* = 5 for *Keap1*^FA/FA^ mice). The data represent the mean ± SEM. *P < 0.05. Unpaired two-tailed Student’s *t*-test was applied. The samples of the WT mice at 5 months of age are the same as those shown in Figs. 1 and 2. Scale bars correspond to 50 μm (**d**, **e**).

At 5 months of age, when elevation of ABR thresholds was already detectable in WT mice (see Fig. 1a), the ABR thresholds of *Keap1*^FA/FA^ mice tended to be lower than those of WT mice, although the difference did not reach statistical significance (Fig. 7a). The ABR wave I latencies were comparable between *Keap1*^FA/FA^ and WT mice as observed at 2 months of age (Fig. 7b). The ABR wave I amplitudes were larger in *Keap1*^FA/FA^ mice than WT mice as observed at 2 months of age (Fig. 7c), although the amplitudes in 5-month-old mice were lower than those in 2-month-old mice in both genotypes (see Fig. 6c). Histological differences were not obvious between *Keap1*^FA/FA^ and WT mice (Fig. 7d, e).

### Protection of the cochleae from age-related degeneration by *Keap1* knockdown

**Fig. 8:**
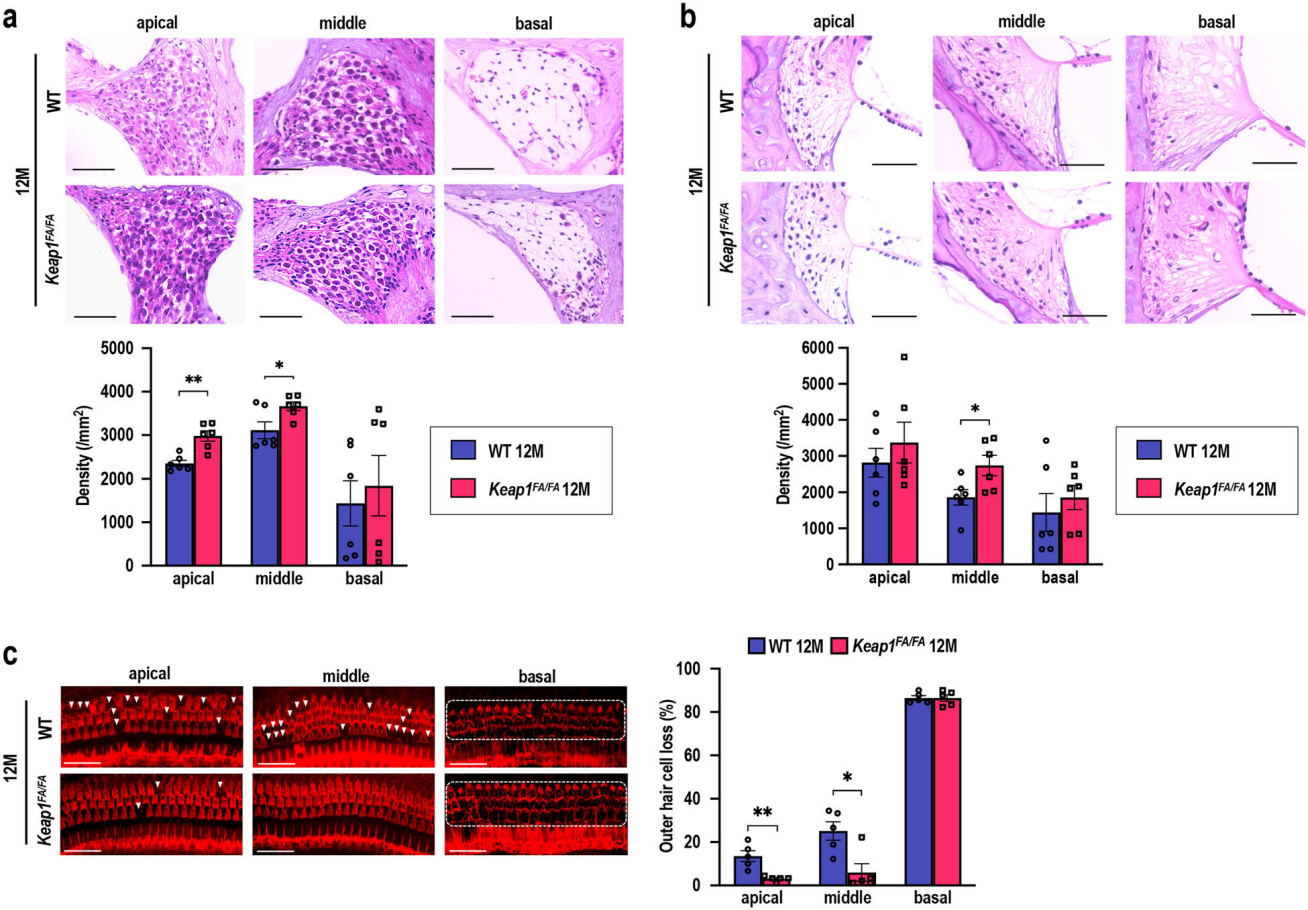
Comparison of cochleae in the WT and *Keap1*^FA/FA^ mice at 12 months of age. **a**, **b** Cochlear histology at each turn. The SGN density (**a**) and density of the SL fibrocytes (**b**) are shown (*n* = 6 in each group). **c** Images of the surface preparation of the hair cells. Missing OHCs are indicated with white arrowheads. Almost all OHCs at the basal turns are missing, as indicated by the area circumscribed by the dashed line. Missing OHCs were quantitatively analyzed by evaluating 90 OHCs at each turn (*n* = 5 in each group). Data represent the mean ± SEM. *P < 0.05, **P < 0.01. Unpaired two-tailed Student’s *t*-test was applied. The samples of the WT mice at 12 months of age are the same as those shown in Fig. 2. Scale bars correspond to 50 μm (**a**, **b**) and 40 μm (**c**).

We then examined histological and morphological alterations in the *Keap1*^FA/FA^ and WT cochleae at 12 months of age, when AHL fully developed in WT mice, in terms of SGN density, SL fibrocyte density, and OHC loss. The SGN density at the apical and middle turns in the *Keap1*^FA/FA^ mice was retained significantly better than it was in the WT mice (Fig. 8a). No differences were apparent in the SGNs at the basal turns in the two mouse genotypes, as the basal SGNs were highly degenerated in the mice of both genotypes. The SL fibrocyte density in the *Keap1*^FA/FA^ mice tended to be higher than it was in the WT mice and the difference in the middle turns reached statistical significance (Fig. 8b). According to the surface preparation images, the OHCs were remarkably well retained in the *Keap1*^FA/FA^ cochleae at the apical and middle turns (Fig. 8c). The OHCs at the basal turns were highly degenerated in both groups. Thus, the morphological and histological integrity of the cochleae at the apical and middle turns was better retained in the *Keap1*^FA/FA^ mice than it was in the WT mice at 12 months of age.

### Attenuation of AHL in the *Keap1*^FA/FA^ mice

**Fig. 9:**
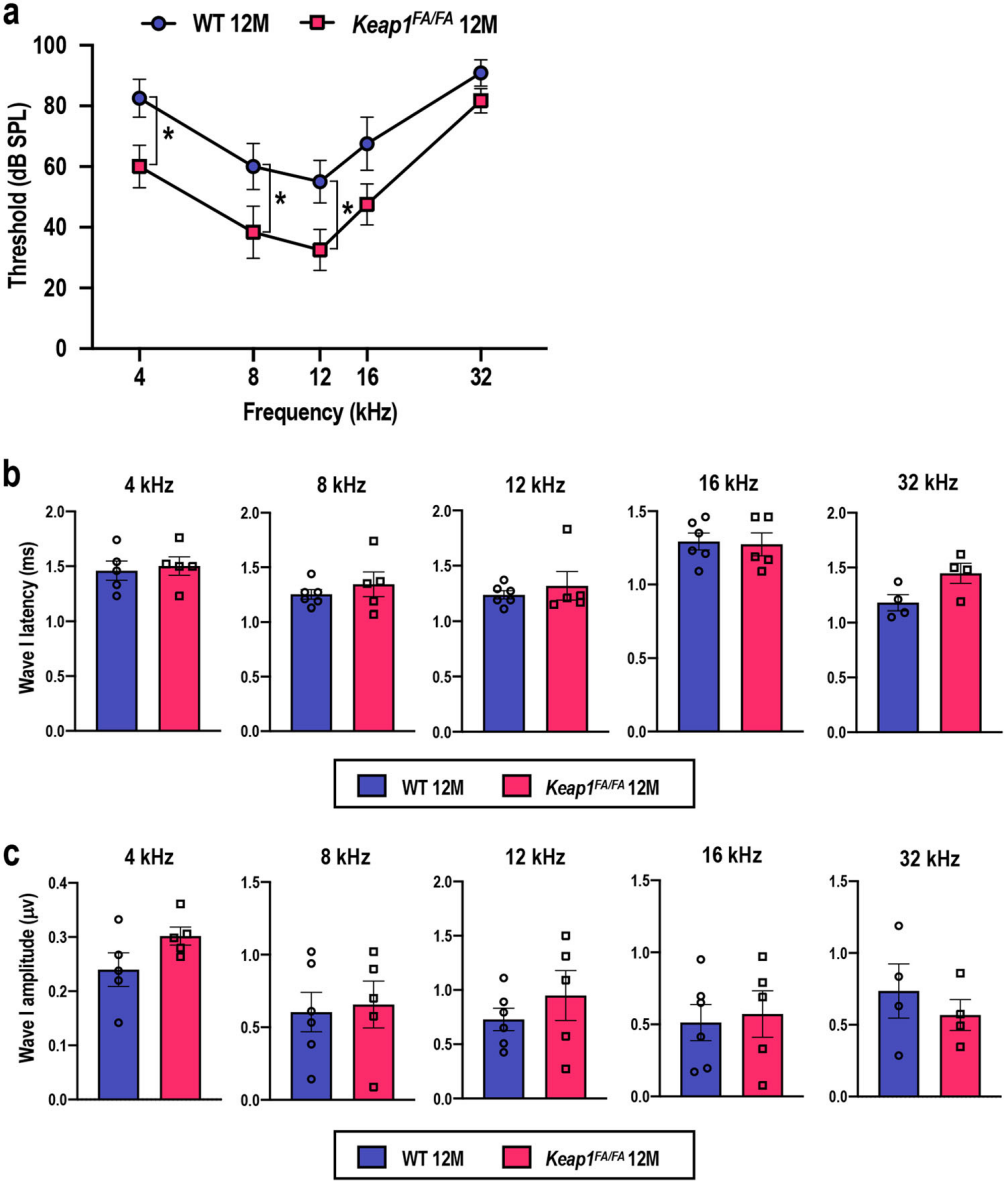
Comparison of ABR of the WT and *Keap1*^FA/FA^ mice at 12 months of age. ABR thresholds (**a**, *n* = 6 for each group), ABR wave I latencies (**b**, *n* = 6 for WT mice and *n* = 5 for *Keap1*^FA/FA^ mice), and ABR wave I amplitudes (**c**, *n* = 6 for WT mice and *n* = 5 for *Keap1*^FA/FA^ mice) of the WT and *Keap1*^FA/FA^ mice at the age of 12 months. One and two WT mice that exhibited ABR thresholds above 100 dB SPL at 4 and 32 kHz, respectively, were omitted from the waveform analysis shown in **b** and **c**. Likewise, one *Keap1*^FA/FA^ mouse that exhibited an ABR threshold above 100 dB SPL at 32 kHz was omitted from the waveform analysis shown in **b** and **c**. The data represent the mean ± SE. *P < 0.05. Unpaired two-tailed Student’s *t*-test was applied. The samples of the WT mice at 12 months of age are the same as those shown in Fig. 1.

To examine whether histological and morphological preservation of *Keap1*^FA/FA^ cochlea resulted in the attenuation of AHL, we measured the ABR thresholds of the *Keap1*^FA/FA^ and WT mice at 12 months of age (Fig. 9a). The ABR thresholds were lower in the 12-month-old *Keap1*^FA/FA^ mice, especially at 4, 8, and 12 kHz, than they were in the 12-month-old WT mice, indicating that hearing ability was better preserved in the *Keap1*^FA/FA^ mice than it was in the WT mice for low- and mid-frequency sounds. Thresholds at 32 kHz were elevated in both the 12-month-old *Keap1*^FA/FA^ and WT mice, indicating that the hearing ability at high frequencies was impaired similarly in the mice of both genotypes. These results are in good agreement with the morphological and histological observations: basal turns that are critical for sensing high-frequency sounds were profoundly degenerated irrespective of *Keap1* status, whereas the apical and middle turns that are critical for sensing low- and mid-frequencies were better preserved in the *Keap1*^FA/FA^ mice than they were in the WT mice.

The ABR wave I latencies and amplitudes were comparable between the two genotypes at 12 months of age (Fig. 9b, c). Of note, the amplitudes, which were larger in *Keap1*^FA/FA^ mice than in WT mice at 2 and 5 months of age, were reduced to the level similar to those of WT mice by 12 months of age, at which age the cochlear histology and ABR thresholds were still better preserved in *Keap1*^FA/FA^ mice than WT mice. As wave I amplitudes are reported to be more sensitive indicators for damages of hair cells and/or SGNs than their histological loss and ABR thresholds^52^, beneficial impacts of *Keap1* inhibition on wave I amplitudes might have disappeared prior to those on histological signs and ABR thresholds. *Keap1*^FA/FA^ cochlea, although delayed, might eventually exhibit functional and structural declines similar to those of WT cochlea.

### Decrease in oxidative stress in the *Keap1*^FA/FA^ cochleae

**Fig. 10:**
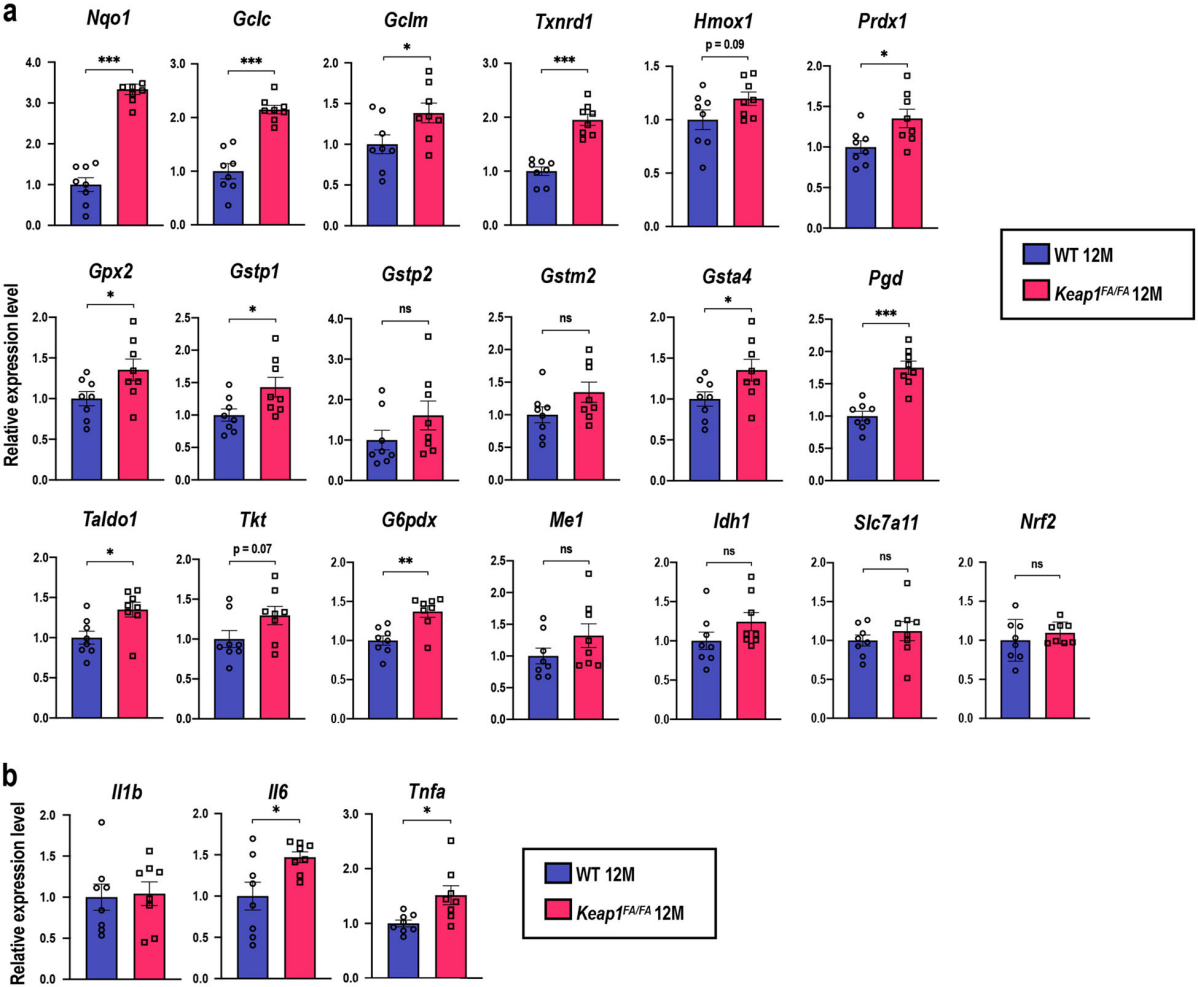
Gene expression in the WT and *Keap1*^FA/FA^ cochleae at 12 months of age. Expression levels of NRF2 target genes and *Nrf2* (**a**), and proinflammatory cytokine genes (**b**) in whole cochleae were evaluated by quantitative real-time PCR (*n* = 8 in each group). All the samples were quantified by using the same standard curve and each expression level was normalized to the *Hprt* or *Beta-Actin* expression level. Average expression levels of WT cochlea are set as 1. The data represent the mean ± SEM. *P < 0.05, **P < 0.01, ***P < 0.001. Unpaired two-tailed Student’s *t*-test was applied. A part of data on the WT mice at 12 months of age are the same as those shown in Fig. 3.

We examined whether NRF2 target genes were elevated in the *Keap1*^FA/FA^ cochleae in mice at 12 months of age (Fig. 10a). Eleven genes out of 18 representative NRF2 target genes were significantly upregulated in the *Keap1*^FA/FA^ cochleae. Expression levels of the remaining seven genes tended to be also higher in the *Keap1*^FA/FA^ cochleae than WT cochleae, but without reaching statistical significance. Thus, NRF2 pathway was regarded to be activated. The expression of proinflammatory cytokine genes was not significantly different between the *Keap1*^FA/FA^ and WT mice or rather increased in the former at 12 months of age (Fig. 10b). Combining this finding with the observation that aging did not induce the proinflammatory cytokine gene expression in the WT cochleae (see Fig. 3b), we surmise that AHL attenuation in *Keap1*^FA/FA^ mice is less attributable to the control of inflammation.

**Fig. 11:**
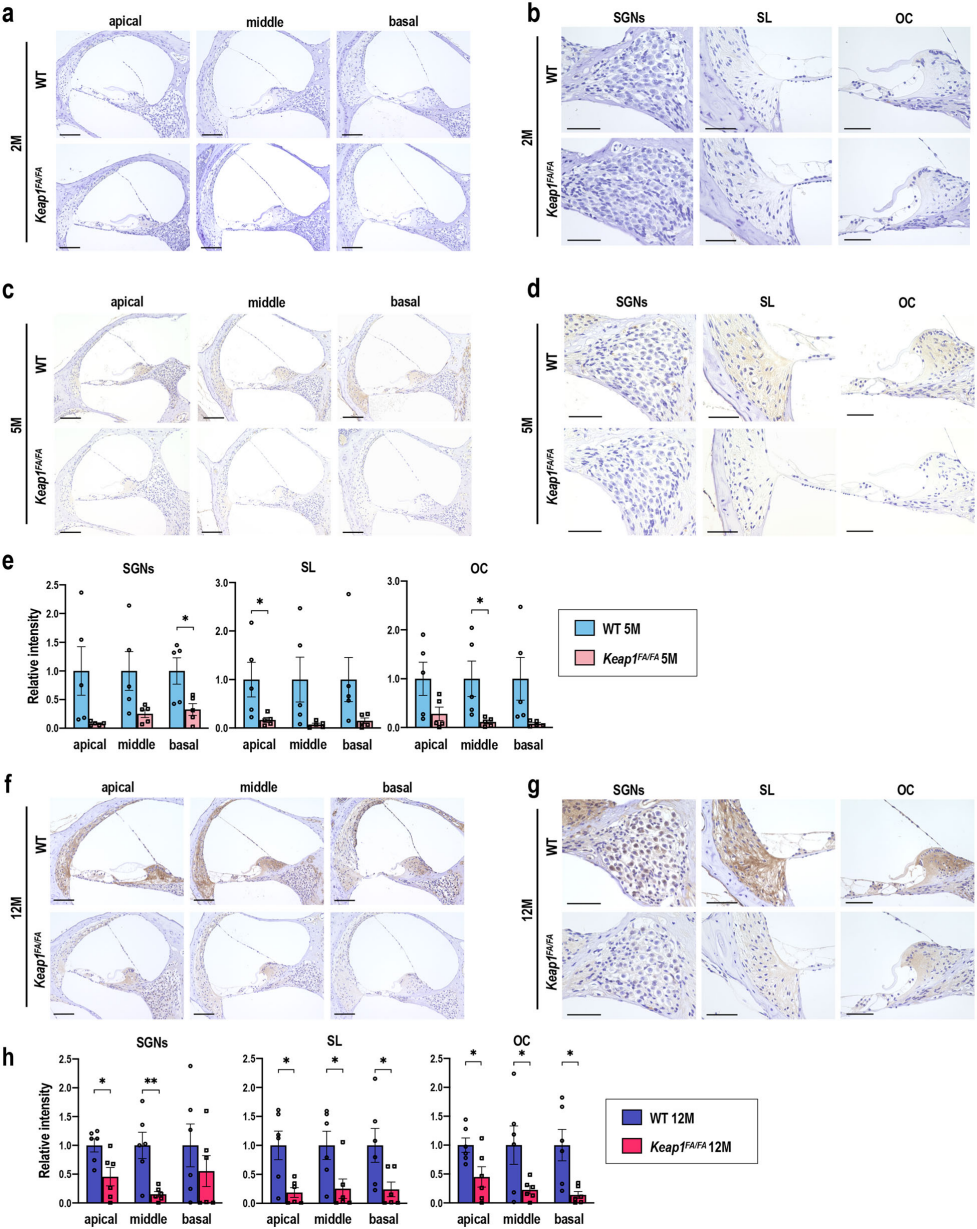
Accumulation of 4-HNE in the WT and *Keap1*^FA/FA^ cochleae during aging. Immunohistochemical staining with 4-HNE in the cochleae at each turn of the *Keap1*^FA/FA^ and WT mice at 2 (**a**), 5 (**c**), and 12 months (**f**). High-power field images are shown for each region (SGNs, SL, and organ of Corti (OC)) in the cochleae at the middle turns in the *Keap1*^FA/FA^ and WT mice at 2 (**b**), 5 (**d**), and 12 months (**g**) of age. The experiments were performed for five samples each of 2- and 5-month-old mice and six samples of 12-month-old mice. Relative intensities of the staining at each turn of 5-month-old cochlea (**e**) and 12-month-old cochlea (**h**) were semi-quantified. Average staining intensities of WT mice are set as 1. The data represent the mean ± SEM. *P < 0.05, **P < 0.01. Unpaired two-tailed Student’s *t*-test was applied. Scale bars correspond to 100 μm (**a**, **c**, **f**) and 50 μm (**b**, **d**, **g**).

As smoldering inflammation was unlikely to be the direct cause of the AHL in the mice used in this study, we hypothesized that NRF2 pathway activation due to *Keap1* knockdown suppressed oxidative stress accumulation and protected the cochlea from the oxidative damage during aging. We examined the accumulation of 4-hydroxy-2-nonenal (4-HNE) as an indicator of oxidative stress in the cochlea by immunohistochemistry. The 4-HNE staining was almost undetectable in both the 2-month-old *Keap1*^FA/FA^ and WT cochleae (Fig. 11a, b). A slight increase in the 4-HNE staining was observed in 5-month-old WT cochlea compared with *Keap1*^FA/FA^ cochlea (Fig. 11c–e). Clear accumulation of 4-HNE was observed in both the 12-month-old *Keap1*^FA/FA^ and WT cochleae but was more remarkable in the latter (Fig. 11f–h). Intense 4-HNE staining was observed in various portions of the cochlea and their intensity levels were nearly similar in all the cochlear turns in the WT mice. 4-HNE accumulation was consistently reduced in various cochlear portions of the *Keap1*^FA/FA^ mice.

**Fig. 12:**
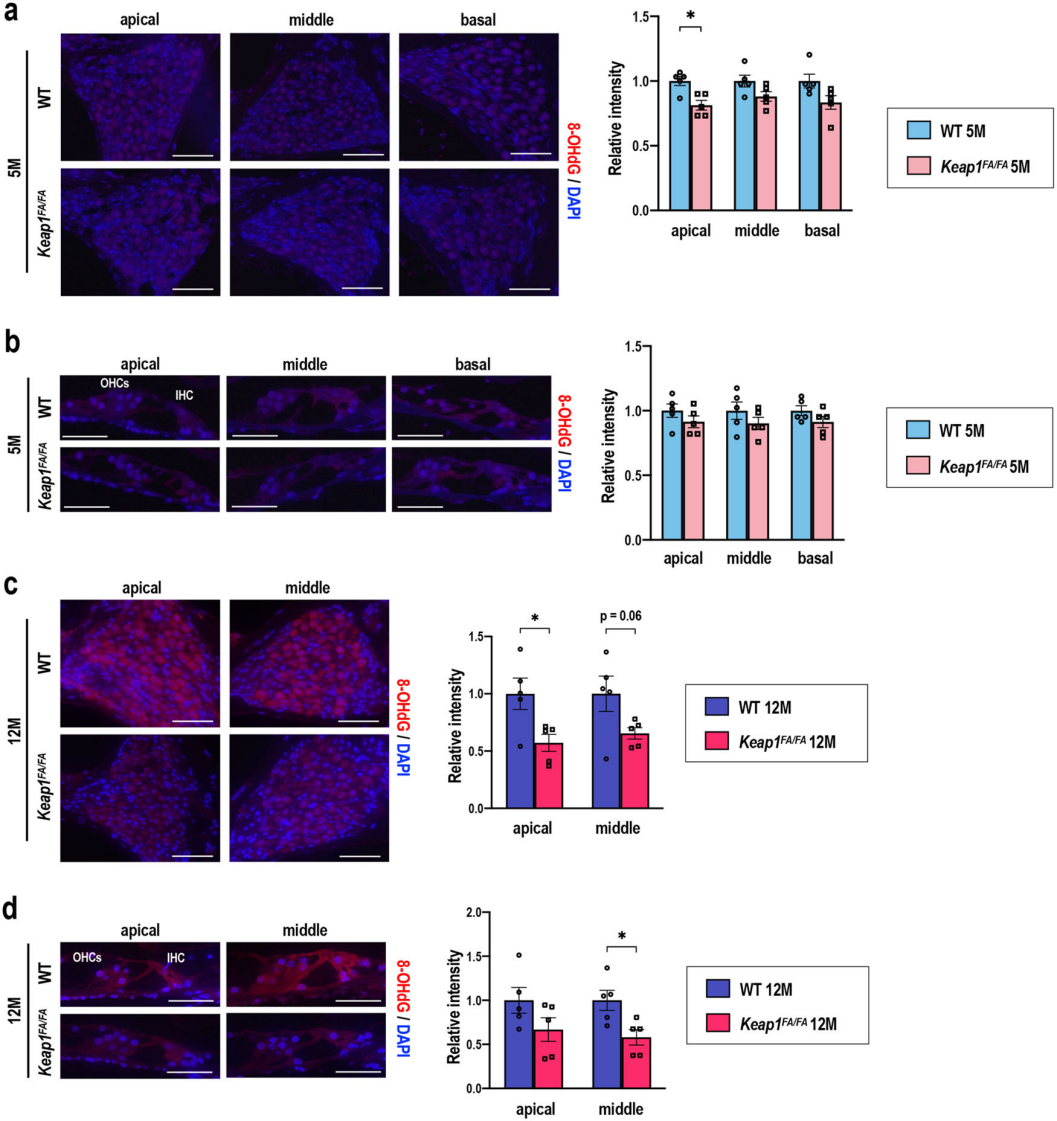
Accumulation of 8-OHdG in the WT and *Keap1*^FA/FA^ cochleae at 5 and 12 months of age. Immunofluorescence detection of 8-OHdG in the SGNs (**a**, **c**) and hair cells (**b**, **d**) at each turn in the *Keap1*^FA/FA^ and WT mice at 5 (**a**, **b**) and 12 (**c**, **d**) months of age. 8-OHdG signals are shown as red fluorescence. Nuclei were counterstained with DAPI (blue). OHCs, outer hair cells; IHC, inner hair cell. The experiments were performed for five samples in each group. Relative intensities of the 8-OHdG signals at each turn were semi-quantified. Average staining intensities of WT mice are set as 1. The data represent the mean ± SEM. *P < 0.05. Unpaired two-tailed Student’s *t*-test was applied. Scale bars correspond to 50 μm (**a**, **c**) and 20 μm (**b**, **d**).

As an alternative oxidative stress marker, the accumulation of 8-hydroxydeoxyguanosine (8-OHdG) was measured by immunofluorescence in the cochleae of the 5- and 12-month-old mice. At 5 months of age, intensity of 8-OHdG was similarly low in the cochleae of both genotypes (Fig. 12a, b). At 12 months of age, intense 8-OHdG staining was detected in the SGNs and hair cells at the apical and middle turns of the WT cochlea, whereas the *Keap1*^FA/FA^ mice exhibited less accumulation of 8-OHdG at the corresponding turns (Fig. 12c, d). The accumulation of 8-OHdG at the basal turns was not obvious, probably because the SGNs and hair cells had mostly degenerated (data not shown). These results suggest that NRF2 pathway activation maintains the functional and structural integrity of the cochlea by suppressing oxidative stress accumulation during aging.

## Discussion

In this study, we found that KEAP1 inhibition was effective for attenuating AHL progression in C57BL/6 mice. Suppression of oxidative stress by KEAP1 inhibition was considered to underlie the alleviation of the age-related functional decline of the cochlea. These results are in a good agreement with previous reports describing oxidative damage significantly related to AHL^19,20^. In particular, OHCs were surprisingly well protected in the *Keap1*^FA/FA^ mice, suggesting that OHCs are susceptible to oxidative stress and that enhancement of antioxidant capacity has a large impact on the maintenance of OHC integrity during aging. Consistently, previous studies that analyzed mouse models subjected to oxidative stress described hearing impairment accompanied by loss of OHCs, as well as SGNs^24,53,54^. Although ROS level and antioxidant response of each cochlear component was not precisely evaluated, our study clearly demonstrated that the enhancement of antioxidant capacity is favorable for antagonizing age-related degenerative changes in the cochleae. Considering the strictly specific relationship between KEAP1 and NRF2, namely all apparent phenotypes caused by KEAP1 inhibition, are reversed by simultaneous NRF2 inhibition^55–60^, it is most likely that NRF2 activation due to *Keap1* knockdown is crucial for the prevention of AHL in the *Keap1*^FA/FA^ mice.

The redox steady state depends on the balance between the rates of ROS production and ROS elimination^61^. Similar to the findings of previous studies^62–65^, our results revealed oxidative stress accumulation in the cochleae of the old mice, in which the ROS production level is thought to override the ROS elimination levels. It has been the consensus that, during aging, mitochondrion-derived ROS production increases due to the functional impairment of the mitochondria^64,66,67^. In contrast, changes in ROS elimination caused by aging are likely to vary depending on the tissues and contexts. Antioxidant defense activities were reported to decrease in cochlea and brain during aging^68,69^; however, other reports showed that antioxidant defense activities in the muscle and brain remained unchanged or increased^65,70–72^. In this study, we found that NRF2 pathway activity was not changed in the cochleae for as long as 12 months. We surmise that ROS accumulation during physiological aging is not sufficient to induce NRF2 pathway activation. This supposition explains the reason that active induction of NRF2 pathway by KEAP1 inhibition is effective for the attenuation of AHL.

Notably, NRF2 pathway activation has a dominant protective effect on the apical and middle turns of the cochlea during aging. Currently, mechanisms are unknown how enhanced NRF2 pathway activity exerts differential impacts on each cochlear turn. One of the possible explanations would be that NRF2 pathway may be more active at the apical and middle turns than the basal turns, considering analogy with SOD2 whose antioxidant activity was shown different among cochlear turns^73^. SOD2, which is involved in the NRF2-independent antioxidant pathway, is expressed in the SGNs and their expression levels were found to be higher at the apical and middle turns than it was at basal turns. Antioxidant defense capacities might be generally limited in the basal turns of cochleae. Another possibility is that the basal turns may be affected by molecular mechanisms different from those of oxidative stress, such as mechanical stress. A previous study clearly showed that *Cdh23* SNP inherent to C57BL/6 strain of mice is a cause of the early onset of AHL particularly at high frequencies^24^. As CDH23, a product of *Cdh23* gene, is a component of tip link in stereocilia controlling mechanoelectrical transduction by hair cells^74^, the *Cdh23* SNP is likely to augment mechanical stress, especially in basal turns, which is responsible for sensing of high-frequency stimuli. The impact of antioxidant capacity enhancement by NRF2 pathway activation might be thus diminished in basal turns.

In humans, auditory thresholds typically increase from high frequency at the beginning of AHL, followed by hearing impairment at low–mid frequencies^3,20^. As auditory capacity at mid-frequency is critical for conversation comprehension, preventing the progression of the initial stage AHL, before it advances toward the stage with mid-frequency loss, has a large impact on the quality of life of elderly people. NRF2 activation was shown to be effective for the prevention of NIHL^35^. Administration of an NRF2 inducer, or a KEAP1 inhibitor, before noise exposure, but not after exposure, successfully prevented NIHL. This result implies that early intervention with NRF2 inducers is favorable for the effective prevention of AHL. We propose to start medication for AHL with NRF2 inducers from its initial stage, which is expected to protect the apical and middle turns of the cochlea effectively to maintain auditory capacity at low–mid frequencies. Although currently available NRF2 inducers are all KEAP1 inhibitors, NRF2 could be also activated independently of KEAP1 function by a compound that directly bound to the N terminus of NRF2 and hindered the interaction with KEAP1. To explore and make use of such a compound, further verification is required for the substantial contribution of NRF2 to the prevention of AHL, because we could not obtain a direct evidence of NRF2 nuclear translocation in the *Keap1*^FA/FA^ cochlear cells in this study due to the technical difficulty. Simultaneous disruption of *Nrf2* gene in *Keap1*^FA/FA^ mice will overcome this limitation and give us the answer in the future study.

## Methods

### Animals

Male WT and *Keap1*-knockdown (*Keap1*^FA/FA^) mice^38,39^ on a C57BL/6 genetic background were used in this study. *Keap1*^FA^ is a hypomorphic allele due to the insertion of loxP sequences. *Keap1*^FA/FA^ mice are homozygous for this hypomorphic allele. Mice were genotyped by PCR using the following primers: *Keap1*^FA^ forward, 5′-CAG CAG TTA AGG GCA CCA ATG C-3′ and *Keap1*^FA^ reverse, 5′-CCT GCC TCA GCT TCC CAT CA-3′. The mice were maintained on a normal diet with water ad libitum and housed under a standard 12 h light/12 h dark schedule. All the mice were treated in accordance with guidelines presented in The Standards for Human Care and Use of Laboratory Animals of Tohoku University and Guidelines for Proper Conduct of Animal Experiments by the Ministry of Education, Culture, Sports, Science, and Technology of Japan. The mouse experiments were approved by Institutional Laboratory Animal Care and Use Committee of Tohoku University and Safety Committee for Recombinant DNA Experiments of Tohoku University.

### Hearing function test

The mice were anesthetized using ketamine (100 mg/kg body weight) and xylazine (20 mg/kg body weight) by intraperitoneal administration. ABR recording was conducted using a TDT System 3 auditory-evoked potential workstation and BioSigRP software (Tucker-Davis Technologies). ABR responses were evoked using bursts of pure tones at frequencies of 4, 8, 12, 16, and 32 kHz. Evoked responses were averaged across 1000 sweeps. The responses were recorded for each stimulus level in 5 dB steps from 100 dB SPL to 10 dB SPL. The ABR threshold was defined as the lowest sound intensity sufficient to elicit at least one peak against the averaged ABR value.

The ABR wave I amplitude was determined by measuring the voltage difference between the highest positive value and lowest negative value for the first wave at 100 dB SPL. The ABR wave I latency was measured as the length of time between the onset of the stimulus and the peak of the first wave at 100 dB SPL.

### Histological analysis

Cochleae were quickly dissected from the skull and immediately soaked in 4% paraformaldehyde (PFA). Small holes were made at the round window, oval window, and apex of the cochleae. The cochleae were fixed with 4% PFA at 4 °C overnight and then decalcified in 10% EDTA for 2 days at 4 °C. The decalcified cochleae were embedded in paraffin and 3 μm coronal sections were made. These sections were stained with hematoxylin and eosin, and were visualized using a light microscope (BZ-9000, Keyence, Osaka, Japan). Three cochlear turns (apical, middle, and basal) were used for histological evaluation of each cochlea. Three sections per animal were used for calculation of the mean numbers. Area measurements and cell counts were performed using BZ-H1C software (Keyence).

### Hair cell count

For counting of hair cells, surface preparations of the organ of Corti and the basilar membrane were prepared. The hair cells were stained for F-actin with rhodamine-conjugated phalloidin (1 : 100, Invitrogen) at room temperature for 30 min under light-protected conditions. High-power fluorescence images were obtained using a microscope (BZ-9000, Keyence) and BZ-H1C software (Keyence). The quantitative results were obtained by evaluating 90 OHCs for each turn in a given microscopic field.

### Quantitative real-time PCR analysis

The cochleae were quickly dissected from the skull, placed on ice, and stored at −80 °C. The cochleae were homogenized in ISOGEN (Nippon gene) and, for each sample, the total RNA was purified from whole cochleae. cDNA was synthesized using reverse transcriptase (ReverTra Ace, Toyobo). Quantitative PCR was performed on a QuantStudio3 sequence detector system using Thunderbird SYBR qPCR mix (Toyobo) for the SYBR green system and Thunderbird probe qPCR mix (Toyobo) for the TaqMan probe system. The primers utilized in this study are shown in Supplementary Table 1.

### Immunohistochemistry

Cochlear samples were prepared using a procedure similar to that used for the histological analysis. The decalcified cochleae were embedded in paraffin and 3 μm coronal sections were made. Immunohistochemistry with anti-4-HNE antibody was performed using a Histofine^®⃞^ mouse stain kit (#424021, Nichirei). Tissue sections were deparaffinized, and endogenous peroxidase was blocked by 3% hydrogen peroxide/phosphate-buffered saline (PBS). Sections were subsequently blocked with 10% rabbit serum for 10 min at room temperature and incubated overnight with a primary antibody (anti-4-HNE; 1 : 200, MHN-020P, JaICA) at 4 °C. All sections were washed three times in PBS and incubated with a secondary biotin-conjugated antibody (anti-mouse IgG in Histofine^®⃞^ mouse stain kit; 1 : 500) for 10 min at room temperature. Then, the sections were incubated with peroxidase-conjugated streptavidin and the 3,3′-diaminobenzidine (DAB) substrate was added. Hematoxylin was used for counterstaining.

Immunofluorescence was performed with an anti-8-OHdG antibody. After deparaffinization, we conducted antigen retrieval by autoclaving for 10 min at 121 °C. The tissue sections were blocked with 1% fetal bovine serum (FBS)/PBS for 30 min at room temperature and incubated overnight with a primary antibody (anti-8-OHdG; 1 : 200, bs-1278R, Bioss) diluted in 1% FBS/PBS at 4 °C. All the sections were subsequently incubated with a secondary antibody (Alexa Fluor 594 anti-rabbit; 1 : 300, A11012, Thermo Fisher Scientific) for 1.5 h at room temperature in light-protected conditions. The nuclei were counterstained with 4′,6-diamidino-2-phenylindole (1 : 1000).

Semi-quantification of 4-HNE staining intensity was conducted using ImageJ Fiji software as described previously^75^. Color deconvolution was applied to images, which were converted to black and white images. Subsequently, the maximum threshold was adjusted so that background signal was removed, and DAB signals were measured and expressed as the intensity per area of the cochlear section from each cochlear turn. Immunofluorescence intensity of 8-OHdG was measured using a BZ-9000 microscope equipped with Dynamic cell count software BZ-H1C (Keyence).

### Statistical analysis

All data are presented as the mean ± SEM. Student’s *t*-test and two-way analysis of variance followed by Tukey’s multiple comparison test were utilized. For all tests, *P*-values of < 0.05 were considered significant.

### Reporting summary

Further information on research design is available in the Nature Research Reporting Summary linked to this article.

## Supplementary information

reporting summary

Supplementary Table S1

## Data Availability

The data generated or analyzed during this study are available from the corresponding author on request. *Keap1*^FA/FA^ mice used in this study are available from RIKEN BRC (https://knowledge.brc.riken.jp/resource/animal/card?brc_no=RBRC09595&__lang__=en).
