# Supplementary material for: Activation of the NRF2 pathway in Keap1-knockdown mice attenuates progression of age-related hearing loss
Source: NPJ Aging Mech Dis. 2020 Dec 14;6:14. doi: 10.1038/s41514-020-00053-4 (PMC7736866; doi:10.1038/s41514-020-00053-4)
Supplement: Supplementary file 1 — reporting summary [file 41514_2020_53_MOESM1_ESM.pdf]

nature research

Corresponding author(s): Hozumi Motohashi

Last updated by author(s): 2020/08/18

# Reporting Summary

Nature Research wishes to improve the reproducibility of the work that we publish. This form provides structure for consistency and transparency in reporting. For further information on Nature Research policies, see our Editorial Policies and the Editorial Policy Checklist.

## Statistics

For all statistical analyses, confirm that the following items are present in the figure legend, table legend, main text, or Methods section.

| n/a | Confirmed | |
|---|---|---|
| ☐ | ☒ | The exact sample size (*n*) for each experimental group/condition, given as a discrete number and unit of measurement |
| ☐ | ☒ | A statement on whether measurements were taken from distinct samples or whether the same sample was measured repeatedly |
| ☐ | ☒ | The statistical test(s) used AND whether they are one- or two-sided<br>*Only common tests should be described solely by name; describe more complex techniques in the Methods section.* |
| ☐ | ☒ | A description of all covariates tested |
| ☐ | ☒ | A description of any assumptions or corrections, such as tests of normality and adjustment for multiple comparisons |
| ☐ | ☒ | A full description of the statistical parameters including central tendency (e.g. means) or other basic estimates (e.g. regression coefficient) AND variation (e.g. standard deviation) or associated estimates of uncertainty (e.g. confidence intervals) |
| ☐ | ☒ | For null hypothesis testing, the test statistic (e.g. *F*, *t*, *r*) with confidence intervals, effect sizes, degrees of freedom and *P* value noted<br>*Give P values as exact values whenever suitable.* |
| ☒ | ☐ | For Bayesian analysis, information on the choice of priors and Markov chain Monte Carlo settings |
| ☒ | ☐ | For hierarchical and complex designs, identification of the appropriate level for tests and full reporting of outcomes |
| ☒ | ☐ | Estimates of effect sizes (e.g. Cohen's *d*, Pearson's *r*), indicating how they were calculated |

*Our web collection on statistics for biologists contains articles on many of the points above.*

## Software and code

Policy information about availability of computer code

| | |
|---|---|
| Data collection | No software was used. |
| Data analysis | No software was used. |

For manuscripts utilizing custom algorithms or software that are central to the research but not yet described in published literature, software must be made available to editors and reviewers. We strongly encourage code deposition in a community repository (e.g. GitHub). See the Nature Research guidelines for submitting code & software for further information.

## Data

Policy information about availability of data

All manuscripts must include a data availability statement. This statement should provide the following information, where applicable:

- Accession codes, unique identifiers, or web links for publicly available datasets
- A list of figures that have associated raw data
- A description of any restrictions on data availability

The data generated or analyzed during this study are available from the corresponding author on request.

# Field-specific reporting

# Life sciences study design

All studies must disclose on these points even when the disclosure is negative.

| | |
|---|---|
| Sample size | 5-6 samples were used for histological, morphological and physiological analyses. 8 samples were used for RT-PCR analysis. The sample sizes are relatively small because wild-type and Keap1 knockdown mice in C57BL/6 genetic background, which were examined in this study, are considered as genetically homogeneous. |
| Data exclusions | No data were excluded. |
| Replication | The results were verified and reproduced in multiple independent mice. |
| Randomization | No randamization was conducted in this study. Mice were categorized according to age and genotype. |
| Blinding | Because parameters obtained in this study are not affected by the intention of investigators, we did not conduct blinding. |

# Reporting for specific materials, systems and methods

We require information from authors about some types of materials, experimental systems and methods used in many studies. Here, indicate whether each material, system or method listed is relevant to your study. If you are not sure if a list item applies to your research, read the appropriate section before selecting a response.

## Materials & experimental systems

| n/a | Involved in the study | |
|---|---|---|
| ☐ | ☒ | Antibodies |
| ☒ | ☐ | Eukaryotic cell lines |
| ☒ | ☐ | Palaeontology and archaeology |
| ☐ | ☒ | Animals and other organisms |
| ☒ | ☐ | Human research participants |
| ☒ | ☐ | Clinical data |
| ☒ | ☐ | Dual use research of concern |

## Methods

| n/a | Involved in the study | |
|---|---|---|
| ☒ | ☐ | ChIP-seq |
| ☒ | ☐ | Flow cytometry |
| ☒ | ☐ | MRI-based neuroimaging |

## Antibodies

| | |
|---|---|
| Antibodies used | 1) anti-4-HNE; MHN-020P, JaICA 2) anti-8-OHdG; bs-1278R, Bioss |
| Validation | 1) anti-4-HNE; MHN-020P, JaICA<br>https://www.jaica.com/e/products_lipid_4hne_ab.html<br>2) anti-8-OHdG; bs-1278R, Bioss<br>https://www.biossusa.com/products/bs-1278r |

## Animals and other organisms

Policy information about studies involving animals; ARRIVE guidelines recommended for reporting animal research

| | |
|---|---|
| Laboratory animals | Wild-type and Keap1 knockdown mice in C57BL/6 mice. 2-, 5- and 12-month-old male mice were used. |
| Wild animals | This study did not used wild animals. |
| Field-collected samples | No field-collected samples were used. |
| Ethics oversight | All the mice were treated in accordance with guidelines presented in The Standards for Human Care and Use of Laboratory Animals of Tohoku University and Guidelines for Proper Conduct of Animal Experiments by the Ministry of Education, Culture, Sports, Science, and Technology of Japan. |

Note that full information on the approval of the study protocol must also be provided in the manuscript.
