# Supplementary material for: Activation of the NRF2 pathway in Keap1-knockdown mice attenuates progression of age-related hearing loss
Source: NPJ Aging Mech Dis. 2020 Dec 14;6:14. doi: 10.1038/s41514-020-00053-4 (PMC7736866; doi:10.1038/s41514-020-00053-4)
Supplement: Supplementary file 2 — Supplementary Table S1 [file 41514_2020_53_MOESM2_ESM.docx]

Supplementary Table 1. Primers and probes for real-time RT-PCR

| Gene | Forward primer (5’- -3’) | Reverse primer (5’- -3’) | Probe (5’-FAM- TAMRA-3’) |
| --- | --- | --- | --- |
| Mouse *Hprt* | CTGGTGAAAAGGACCTCTCG | TGAAGTACTCATTATAGTCAAGGG | ATCCAACAAAGTCTGGCCTGTATCCAAC |
| Mouse *Beta-Actin* (*Actb*) | CGGTTCCGATGCCCTGAGGCTCTT | CGTCACACTTCATGATGGAATTGA | - |
| Mouse *Keap1* | CCCATGAGGCATCACCGTAG | CATAGCCTCCGAGGACGTAG | GGATTACTGTGCACCAGGGCAAG |
| Mouse *Nrf2* (*Nfe2l2*) | CAAGACTTGGGCCACTTAAAAGAC | AGTAAGGCTTTCCATCCTCATCAC | AGGCGGCTCAGCACCTTGTATCTTGA |
| Mouse *Nqo1* | AGCTGGAAGCTGCAGACCTG | CCTTTCAGAATGGCTGGCA | ATTTCAGTTCCCATTGCAGTGGTTTGGG |
| Mouse *Gclc* | ATCTGCAAAGGCGGCAAC | ACTCCTCTGCAGCTGGCTC | ACGGGTGCAGCAAGGCCCA |
| Mouse *Gclm* | TGGAGCAGCTGTATCAGTGG | AAATCTGGTGGCATCACACA | - |
| Mouse *Txnrd1* | AGAAAGTGCTGGTCTTGGATTTTG | ACACGTTCCTCCGAGACCC | TCTGGTCCCAAGAGGAGTCGGTGTG |
| Mouse *Hmox1* | GTGATGGAGCGTCCACAGC | TTGGTGGCCTCCTTCAAGG | CGACAGCATGCCCCAGGATTTGTC |
| Mouse *Prdx1* | ACTCAACTGCCAAGTGATTG | TGGGTGTGTTAATCCATGCC | CTTCTGTGGATTCTCACTTCTGTCATC |
| Mouse *Gpx2* | TGTCAGAACGAGGAGATCCTG | GACTAAAGGTGGGCTGGTACC | CCTCAAGTATGTCCGACCTG |
| Mouse *Gstp1* | GCAAATATGTCACCCTCATCTACACC | GCAGGGTCTCAAAAGGCTTCA | AGGGCCTTCACGTAGTCATTCTTACCATTCTCATAGT |
| Mouse *Gstp2* | CAAATATGGCACCATGATCTACAGA | GCAGGGTCTCAAAAGGCTTCA | AGGGCCTTCACGTAGTCATTCTTACCATTCTCATAGT |
| Mouse *Gstm2* | GTAGGATTACAAAGCCCAGACCTG | AAGAAATGGAGAGCCCAAGGAC | - |
| Mouse *Gsta4* | GGGAACAGTATGAGAAGAAGATGCAAAA | CCCATCGATTTCAACCAAGG | TGCACACCTGCTTTTCGGCCAAG |
| Mouse *Pgd* | ATGCCAGGAGGGAACAAAG | GTTCTCCGGTTCCCACTTTT | - |
| Mouse *Taldo1* | TTATCATCAACCTGGGAGGG | GCGAAGGAGAAAAGCAGTGT | - |
| Mouse *Tkt* | CGAAACCCTCACAATGATCG | TTCCTCAGGTTCAGCAGCTC | - |
| Mouse *G6pdx* | GTCCAGAATCTCATGGTGCTGA | GCAATGTTGTCTCGATTCCAGA | - |
| Mouse *Me1* | GGAGCTCCAGGTCCTTAGAA | TGAGCACGCTGTAGAAGAGC | - |
| Mouse *Idh1* | AAAATATCCCCCGGCTAGTG | TCTCTACTTTTCCAGGCCCA | - |
| Mouse *Slc7a11* | TGGGTGGAACTGCTCGTAAT | AGGATGTAGCGTCCAAATGC | AGCTACTGCTGTGATATCCC |
| Mouse *Il1b* | TGCCACCTTTTGACAGTGATG | TGATGTGCTGCTGCGAGATT | - |
| Mouse *Il6* | CTGCAAGAGACTTCCATCCAG | AGTGGTATAGACAGGTCTGTTGG | - |
| Mouse *Tnfa* | CACGCTCTTCTGTCTACTGAA | GGCTACAGGCTTGTCACTCGA | - |
